# Building the synaptonemal complex: Molecular interactions between the axis and the central region

**DOI:** 10.1371/journal.pgen.1010822

**Published:** 2023-07-20

**Authors:** Spencer G. Gordon, Ofer Rog

**Affiliations:** School of Biological Sciences and Center for Cell and Genome Sciences, University of Utah, Salt Lake City, Utah, United States of America; Stowers Institute for Medical Research, UNITED STATES

## Abstract

The successful delivery of genetic material to gametes requires tightly regulated interactions between the parental chromosomes. Central to this regulation is a conserved chromosomal interface called the synaptonemal complex (SC), which brings the parental chromosomes in close proximity along their length. While many of its components are known, the interfaces that mediate the assembly of the SC remain a mystery. Here, we survey findings from different model systems while focusing on insight gained in the nematode *C*. *elegans*. We synthesize our current understanding of the structure, dynamics, and biophysical properties of the SC and propose mechanisms for SC assembly.

## Introduction

Meiosis is a specialized cellular process essential to sexually reproducing organisms. During meiosis, parental chromosomes (homologs) are packaged, paired, and segregated into gametes. In most organisms, chromosome segregation relies on physical exchanges of genetic information between homologs called crossovers. For crossovers to form, chromosomes are brought together and aligned along their lengths. The way homologs find their partner (“pair”) varies between clades, with some organisms relying on the repair of double-strand breaks by homologous recombination and others pairing homologs independently of breaks. Regardless of the mechanism of pairing, almost all organisms align their homologs end-to-end and bring them in close proximity (a process termed “synapsis”) by assembling a ladder-like structure between them called the synaptonemal complex (SC). The SC is essential for the regulation of almost all meiotic processes [1,2].

**Fig 1:**
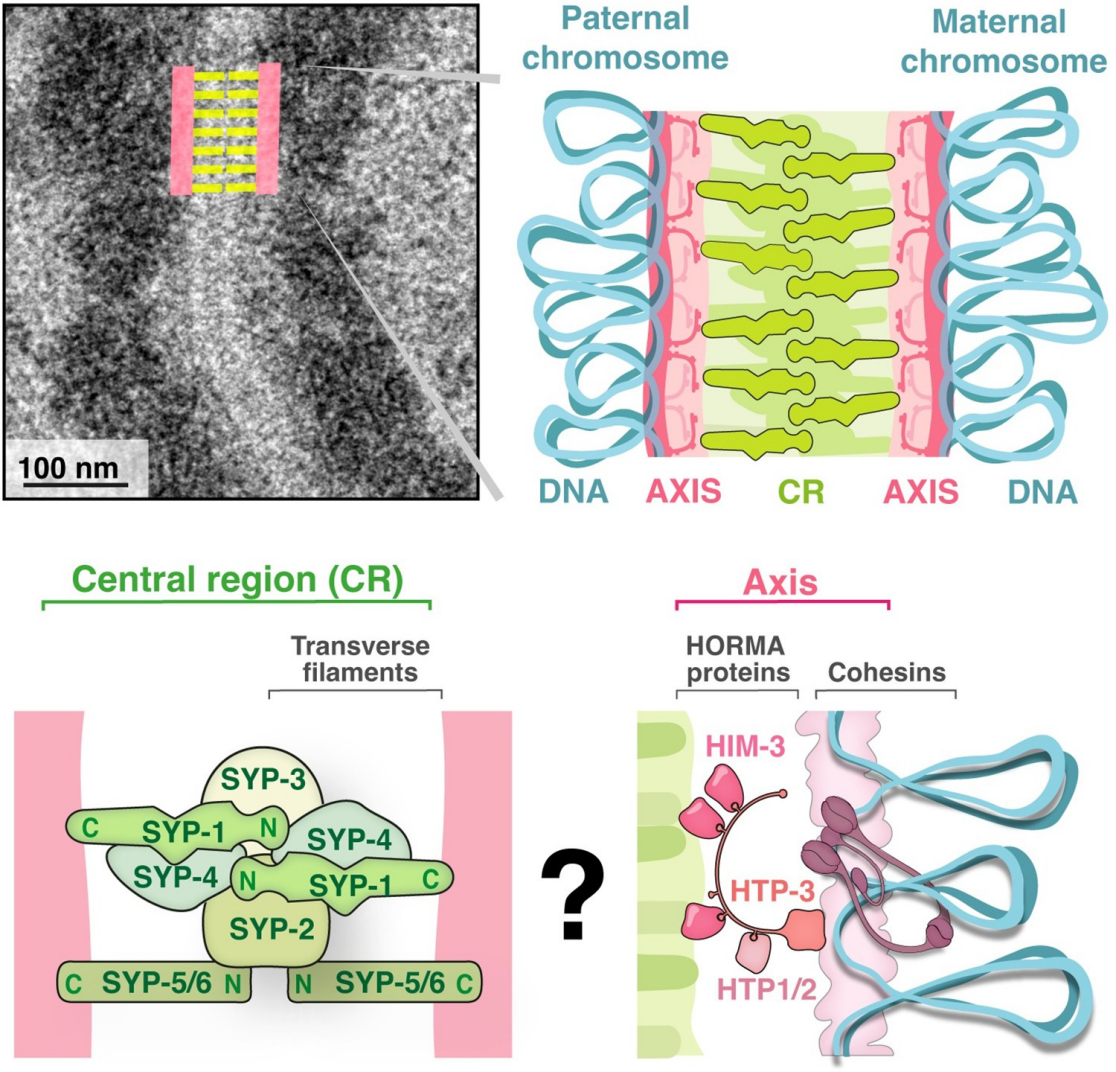
SC structure in *C*. *elegans*. Top: Electron micrograph of meiotic chromosomes in *C*. *elegans* (adapted from [12]). The electron-dense mass to the sides of the SC is chromatin. Axes (salmon) organize each of the parental chromosomes into an elongated structure by stacking the bases of chromatin loops (blue). The CR (green) assembles between the parallel axes of the homologs. Bottom: Magnified views of the CR and the axis. The CR (left) is composed of SYP-1 through SYP-6. The axis (right) is composed of ring-shaped cohesins (mauve) and the HORMA-domain proteins HTP-3 (orange), which, in turn, recruits the HORMA-domain proteins HIM-3 and HTP-1 and HTP-2 (pink). See Table 1 for more details.

The SC is composed of two major components that were initially defined cytologically and subsequently genetically and functionally ([2]; Fig 1). The axis is a stiff filamentous structure that assembles on each homolog individually, organizes chromatin as an array of loops, and acts as a scaffold for chromosomes to interact. (The terms “axial elements” and “lateral elements” have been used to refer to the axis before synapsis and after assembly into the SC, respectively; here, we use the term “axis” to encompass both meanings.) The central region (CR) of the SC assembles between parallel axes and appears as a ladder or railroad track in negative-stained electron micrographs.

While components of the axis and CR have been known since the 1980s (Table 1), a CR-axis interface has not been clearly defined in any system. Here, we review what is known about CR-axis interactions in meiotic model organisms, with a focus on insight from *C*. *elegans*, a model system with detailed genetic, structural, and cytological information. We also contextualize these data with current understanding of the material properties of the CR. We have limited our discussion of the many meiotic functions of the SC and of molecular interactions within the axis and within the CR. These topics have been covered in recent excellent reviews [3–11].

**Table 1:** SC components across model organisms.

organism	central region			axis				references
	transverse filaments	central element	other	HORMA	core	meiotic cohesins*	other	
*C*. *elegans*	SYP-1 SYP-5/6**		SYP-2 SYP-3 SYP-4	HTP-1/2** HTP-3 HIM-3		REC-8 COH-3/4**		[13–24]
*S*. *cerevisiae*	Zip1	Ecm11 Gmc2		Hop1	Red1	Rec8	Mek1	[25–30]
*A*. *thaliana*	ZYP1a/b**			ASY1	ASY3 ASY4			[31–34]
*D*. *melanogaster*	C(3)G	Corona Corolla				C(2)M ORD SOLO SUNN		[35–41]
*M*. *musculus*	SYCP1	SYCE1 SYCE2 SYCE3 TEX12 SIX6OS1		HORMAD1 HORMAD2	SYCP2 SYCP3	REC8 RAD21L STAG3		[42–54]

*For simplicity, we omitted non-meiosis-specific components of the cohesin complex.

**Closely related proteins that are redundant or partially redundant.

### SC structure

#### The axis

The axis consists of a different number of components in different organisms (Table 1) but usually contains at least one HORMA domain-containing protein. The name HORMA describes a conserved fold initially identified in three chromosome-associated proteins in yeast (Hop1 [component of the meiotic axis], Rev7 [subunit of DNA polymerase], and Mad2 [mediating the spindle assembly checkpoint]; [55]). HORMA domains bind to “closure motifs”—short peptides either on the C-terminus of the same protein or on different proteins—which alters their conformation and can facilitate complex formation [5].

The axis also contains two other protein families: cohesins and axis core proteins [6]. Meiosis-specific cohesin complexes mediate both canonical functions, such as sister chromatid cohesion, as well as meiosis-specific roles, such as the removal of cohesion in two regulated steps during the two meiotic divisions [56–58]. Cohesins interact with and topologically entrap DNA [59], suggesting they interact with chromatin to create a scaffold onto which other axis components assemble. Consistent with their interaction with DNA, cohesins in worms localize farthest from the center of the SC [60], and their removal prevents all other axis components from interacting with chromosomes [22]. Axis core proteins have been identified in yeast, plants, and mammals. These proteins contain coiled-coils and can form fibers and fiber bundles in vitro, suggesting they play a role in building the stiff core of the axis [61].

#### The CR

The CR forms between paired chromosomes and appears in negative-stained electron micrographs as a 100- to 150-nm-wide ladder ([1,2]; Fig 1). Although the ultrastructure of the CR is similar in different organisms, the number of components and their primary sequence varies widely ([62,63]; Table 1). Conservation is limited to a few short sequence motifs and to the position and length of coiled-coils [62,64]. Despite this sequence divergence, CRs contain at least one “transverse filament”—a protein that spans the width of the CR in an N-terminal head-to-head orientation and that has long coiled-coils that help determine the distance between the axes [65–67].

In some species, an electron-dense band called the central element runs along the middle of the CR. Some CR components localize to this region (Table 1) and, based on their mutant phenotype, are suggested to promote the elongation of the SC from its initial assembly sites to the rest of the chromosome [25,68].

### CR-axis interactions across meiosis model organisms

The CR forms the SC by interacting with the axes, which serve as a surface for its assembly. However, CR-axis interaction is not trivial in two respects. First, the CR only associates with paired axes—unpaired axes tend not to associate with CR material (e.g., [69]). Second, the CR does not strictly require the axis to self-associate. When it cannot assemble onto chromosomes, either due to lack of homolog pairing (i.e., absence of a two-axis substrate) or if the axis is genetically perturbed, CR material forms aggregates called polycomplexes, which ultrastructurally resemble stacked CR segments [70–72]. In some organisms, polycomplexes form in unperturbed meiosis, usually before SC assembly or after SC disassembly [72]. Nonetheless, the CR shows a preference for paired axes over polycomplex formation [12,73]. A possible interpretation of this observation is that paired axes help overcome a nucleation barrier for the CR (see below).

In some organisms, the composition of the axis changes upon synapsis. In yeast, plants, and mammals, the HORMA domain-containing axis components are evicted during synapsis, albeit to differing degrees [46,74,75]. HORMA protein removal involves the conserved ATPase Pch2/TRIP13, which can alter the conformation of HORMA domain proteins [6,11,76,77]. This raises the possibility that HORMA proteins are antagonistic with the CR, either directly or by masking CR-interacting surfaces on other axis proteins. An alternative possibility is that an assembled CR promotes the eviction of HORMA proteins, e.g., by recruiting Pch2/TRIP13 [78].

Most of our knowledge of the SC has been gained through molecular genetics and cytology. These tools were able to identify many SC components and localize them within the SC. However, many null mutations in single subunits prevent SC assembly altogether. In addition, the multiple functions of the SC (and, hence, the pleiotropic effects of mutations) have made it difficult to assign distinct functions to many proteins and domains. These limitations have made it challenging to identify alleles that are consistent with specific perturbations of CR-axis interactions. Only in a few model systems informative mutations have been identified, e.g., point mutations or small truncations that prevent SC assembly but allow both axis assembly and formation of polycomplexes by the CR material [79,80]. However, even in these cases, the phenotypes may be the result of perturbing other meiotic processes, like pairing. Lacking an established in vitro reconstitution system to study the SC, further validation and refinement of such candidate CR-axis interaction interfaces have proven tricky.

While an axis-CR interface has not been defined in any model system, we discuss below experimental evidence from meiosis model organisms that points to candidate proteins or domains, first in the axis and then in the CR.

#### *Saccharomyces cerevisiae* (budding yeast)

The axis in budding yeast consists of cohesins [58], the core protein Red1 [27,61], the kinase Mek1 [28], and the HORMA protein Hop1 [30]. The HORMA domain in Hop1 binds a closure motif on Red1 [81]. In the absence of either Red1 or Hop1, SC formation is perturbed and polycomplexes form, although the SC in these mutants retains some chromosome association [65,82,83]. Red1 contributes to CR-axis interactions: It weakly interacts with the CR transverse filament protein Zip1 in a yeast two-hybrid assay, and this interaction is much stronger upon SUMOylation of Red1 [82,84]. Nonetheless, overall SC assembly is slowed, but not abolished, in mutants in which the Red1-Zip1 interaction is abolished [85], suggesting other components contribute to CR-axis interaction. Hop1 protein sequence includes a DNA and chromatin interaction domain [86,87], placing it farther away from the CR. Hop1 is also partially evicted from the axes upon synapsis [76]. Rec8, although it localizes to chromatin and, therefore, away from the CR, might contribute to CR interaction based on the observation that Rec8 phospho-mutants exhibit SC assembly phenotype despite not discernably affecting Hop1 or Red1 localization [88,89].

Zip1 assembles in a head-to-head orientation that places the C-terminus closest to the axis, making its C-terminus a candidate to mediate CR-axis interactions [90]. Consistent with this, upon deletion of the last 30 a.a. of Zip1, the CR does not assemble between chromosomes and instead forms polycomplexes [79]. The last 30 a.a. of Zip1 are also responsible for the abovementioned yeast two-hybrid interactions with Red1 [84].

#### *Mus musculus* (mice)

The axis in mammals is made up of four proteins in addition to cohesins, two of which include a HORMA domain (HORMAD1 and HORMAD2; [46]). These HORMA domain proteins are depleted during synapsis, suggesting they are not the major interactors with CR. Nonetheless, HORMAD1 is crucial for proper SC assembly [46,91–93]. The other two axis components are SYCP2 and SYCP3 [53,54]. SYCP3 is necessary for axis localization of SYCP2 [61]. The C-terminus of SYCP2 interacts with the CR component SYCP1 in vitro and in yeast two-hybrid, and SYCP2 is recruited to SYCP1 polycomplexes when the two proteins are expressed in the cytoplasm of non-meiotic cells [94]. However, when either SYCP2 or SYCP3 are deleted, CR components form aberrant SC but not polycomplexes [95,96], suggesting it is not only SYCP2 that is mediating axis-CR interaction.

The transverse filament in the mouse CR, SYCP1, assembles in an N-terminal head-to-head orientation that spans the width of the CR [67,97,98]. The C-terminus of SYCP1 is responsible for the abovementioned yeast two-hybrid interaction with the axis component SYCP2 [94]. Notably, the C-terminus of the human SYCP1 interacts with naked DNA in vitro [99], suggesting that non-axis-mediated interactions also contribute to SC assembly.

#### *Arabidopsis thaliana* (plants)

ASY1, a HORMA axis protein, is depleted from axes as synapsis occurs [75]. The other main component of the axis is ASY3 [33]. When ASY3 is deleted, ASY1 is unable to assemble normally onto chromosomes, and the CR fails to assemble an SC and instead forms polycomplex-like structures. These phenotypes, as well as the observation that ASY3 is part of the assembled SC, point toward ASY3 as the axis component involved in CR-axis interactions.

*A*. *thaliana* contains two highly similar transverse filament CR components, ZYP1a/b, which are functionally redundant [31,100,101]. ZYP1a/b assembles in a head-to-head fashion, with the N-terminus in the middle of the CR and the C-terminus closest to the axis [31].

#### *Drosophila melanogaster* (flies)

The axis cohesin subunit C(2)M colocalizes with the C-terminus of C(3)G [35,102]. When C(2)M is deleted, the CR cannot form between chromosomes [39]. However, C(3)G still appears chromosome-associated in the absence of C(2)M and does not form canonical polycomplexes, suggesting residual axis or chromosomal associations. Cohesins are likely responsible for these residual interactions. While the axis component ORD is not essential for CR-axis association [38], removing both C(2)M and ORD or removing cohesins altogether (which prevents C(2)M assembly) eliminates C(3)G chromosome association [103]. So far, no HORMA domain-containing axis proteins have been identified.

The C-terminus of the transverse filament CR component C(3)G colocalizes with the axis, whereas its N-terminus localizes to the middle of the SC [66,102,104]. Deletion of the C-terminus of C(3)G prevents loading of the CR onto chromosomes, which instead form polycomplexes, suggesting it is necessary for axis association [80].

### CR-axis interactions in nematodes

#### Candidate axis components to mediate CR-axis interactions

Removal of the axis protein HTP-3 (which is required for the other HORMA proteins to associate with chromosomes; [19]) or of cohesins (which are required for all other axis proteins to interact with chromosomes) abolish CR-axis interactions, based on the formation of spherical polycomplexes that do not interact with chromosomes [22]. Upon cohesin deletion, other axis components are not recruited onto chromosomes and instead colocalize with polycomplexes. However, when HTP-3 is deleted, the other axis components do not localize to polycomplexes (or to chromosomes). This suggests that HTP-3 is a main interactor with the CR. However, HIM-3 and HTP-1 cannot be detected by immunofluorescence when HTP-3 is deleted despite the presence of *him-3* mRNA, suggesting they require HTP-3 for their stability [19,22]. Deletion of the closure motifs on HTP-3 that recruit HIM-3 to the axis causes a similar absence of HIM-3 [105]. Further support for the idea that it is not HTP-3 that interacts with the CR is provided by the analysis of *htp-3*^*6GK*^, in which all six closure motifs are mutated. In *htp-3*^*6GK*^ animals, only HTP-3 assembles onto chromosomes and the CR forms spherical polycomplexes that do not associate with chromatin [105].

Several lines of evidence suggest the axis protein HIM-3 is a candidate to be the main CR interactor. Super-resolution microscopy places HIM-3 as the closest axis component to the CR [13,60,106]. Successive elimination of the four HIM-3-recruiting closure motifs on HTP-3, which reduces the amount of HIM-3 on the axis, results in gradually increasing defects in SC assembly [107]. Finally, *him-3* hypomorphs (*vv6* and *me80*) that have been suggested to destabilize HIM-3 result in fewer synapsed chromosomes [108].

While HIM-3 may be the axis component responsible for most CR-axis interactions, it is likely that HTP-1 and HTP-2 also harbor a certain affinity for the CR. In *him-3* null worms, the CR forms thick, elongated assemblies. However, their non-spherical shape compared with *htp-3* null polycomplexes suggests that CR material maintains loose associations with unpaired axes, which contain HTP-1 and HTP-2 [108]. In *htp-1* and *htp-1/2* null animals, synapsis is affected but is not eliminated [18,22,24], suggesting HTP-1 and HTP-2 carry a regulatory role in SC assembly, rather than being the main CR interactors. This idea is further supported by the observation that it is diffuse, rather than axis-associated HTP-1 that regulates synapsis [109].

Large-scale removal of axis subunits upon SC assembly, which could provide hints on CR-axis interaction, has not been documented in worms (e.g., [108]). However, CR disassembly in worms offers potential clues. At the end of meiosis, the CR disassembles in two stages [110]. Initially, each chromosome is partitioned into two “arms,” one on each side of the single crossover, and the CR is depleted from the longer of these two chromosomal arms. Later, the CR is depleted from the rest of the chromosome. Concomitant with CR partitioning to the short arm, HTP-1/2 are partitioned to the long arm, while HIM-3 and HTP-3 remain on both arms [110,111]. The seeming repulsion of the CR away from HTP-1/2 and towards an arm that only contains HIM-3 and HTP-3 could indicate that the CR has a higher affinity for HIM-3 and HTP-3 and a weaker affinity for HTP-1/2.

#### Candidate CR components to mediate CR-axis interactions

The CR components involved in CR-axis interactions have proven more elusive. The main reason for this is the non-informative phenotype of null mutations in the six nematode CR proteins, which lead to a complete absence of the CR [15–17,23]. In addition, the partially redundant SYP-5/6 were only recently identified [13,14].

The C-termini of the transverse filament proteins SYP-1 and SYP-5/6 are closest to the axis, supporting them as candidate axis interactors [13,106,112]. While worm SC does not have a cytologically discernable central element, SYP-2 and SYP-4 were shown to localize to the center of the CR via immuno-EM and super-resolution microscopy [106,112]. Data for SYP-3 are conflicting, with immuno-EM suggesting SYP-3 is close to the axis, while immuno-labeling of tagged protein in super-resolution microscopy placed it in the middle of the CR [106,112]. By virtue of their localization, these CR components are unlikely to physically interact with the axis.

Attempts to generate hypomorphs in CR components have so far failed to yield mutations consistent with specific disruption of CR-axis interactions, e.g., mutations that cause CR material to form polycomplexes. Truncation analysis of SYP-5/6 revealed that the more of the C-terminus that is removed the fewer chromosomes synapse [13]. While consistent with a role in CR-axis interactions, this phenotype could also stem from other perturbations to the SC, like a reduction in intra-CR interactions. Consistent with the latter idea, the C-termini of SYP-5/6 interact with other CR components [14].

### Characteristics of SC assembly in nematodes

#### Two-stage assembly

The SC forms in two distinct stages. The first is a localized assembly, akin to nucleation. Nucleation depends on the process of pairing, where homologous sequences on the two parental chromosomes are brought together. In worms, nucleation is rate limiting for the completion of synapsis [73]. Chromosomes that fail to pair do not nucleate SC assembly and remain asynapsed, as is observed for the sex chromosomes of heterogametic sexes. In worms, this is also observed in response to perturbation of the *cis*-acting sites that mediate pairing or the proteins that bind them [69,113], and in triploid and aneuploid animals with three copies of the X chromosome [114]. Related observations were made in other model organisms (e.g., [115–117]).

The second stage of SC assembly is the processive elongation of the SC along the chromosome. Elongation is swift, progressing at 150 nm/min and synapsing the entire chromosome in approximately 30 minutes ([73]; similar observations have been made in budding yeast [118]). Elongation appears to be mostly sequence homology independent. The SC can assemble between non-homologous sequences, including in worms heterozygous for translocations and chromosome fusions [69], and when chromosomes fold back on themselves and synapse their left and right arms [119,120]. At least in one scenario where the SC assembles between non-homologous chromosomes—in *htp-1* mutants—synapsis proceeds as fast as between homologs [73]. Finally, synaptic adjustment, where already-assembled SC rearranges to eliminate junctions and equalizes the lengths of the axes to minimize asynapsed regions, has been observed in worms and many other organisms [117,121] and is also consistent with sequence-independent SC assembly.

#### Coupling SC assembly to chromosome alignment

The two stages of SC assembly have different relationship to axis alignment. Nucleation depends on the prior proximity of the two axes, which are brought together by the machinery that mediates pairing [113]. Elongation, however, rather than requiring the axes to be paired, is helping to bring the axes together. In worms lacking a CR, the axes are mostly splayed apart and are held together only at the site of pairing, where nucleation would occur [23,122]. Even in organisms where the homologs are aligned prior to SC assembly, like budding yeast or *Sordaria*, SC elongation helps to bring the axes into close juxtaposition (approximately 100 nm; [117]).

The energy for the apparent work of bringing chromosomes together could come from two sources. The first may be a Brownian ratchet [123]: Thermal fluctuations of the two axes, constrained through nearby CR-dependent tethering, are stabilized by further extension of the CR. This process is dramatically accelerated by chromosome motions mediated by cytoskeletal-associated motor proteins—a conserved feature of meiosis [1,124–126]. In worms, the rate of SC elongation is 6-fold slower when the chromosome-cytoskeleton attachment is perturbed (150 *versus* 25 nm/min in wild type versus mutant, respectively; [73]), giving a rough estimate of the relative contributions of these two mechanisms.

#### Cooperative assembly

The ability of the CR to assemble onto chromosomes is sensitive to CR subunit concentration. Down-regulation using RNAi showed that SC assembly is tolerant of approximately 50% reduction in CR subunit concentration, but once the concentration is reduced by >70%, many chromosomes fail to synapse [127,128]. Importantly, the few chromosomes that are CR associated in the latter condition assemble SC along their entire length. This result suggests that nucleation and/or elongation involve cooperative assembly: When CR subunit quantities are limiting, the CR tends to assemble along an entire chromosome more readily than loading onto additional chromosomes. The affinity of CR to itself is also evident by the accumulation of CR material on already-synapsed chromosomes ([12,129]; similar observations were made in yeast [130]).

#### Liquid-crystalline properties of the CR

Although the CR appears as a highly organized ladder in electron micrographs [131], recent work has revealed that it has properties of liquids. Components of the CR (but not the axis) are dynamic, the CR seems to flow to one side of the chromosome during disassembly, and CR polycomplexes exhibit typical liquid behaviors like structural deformation, fusion, and resorption [12,14,129,132]. These observations suggest that the CR has both liquid and crystalline properties. These liquid characteristics may underlie the difficulty in defining a conventional protein–protein interface between the CR and axis, since condensates form through weak multivalent interactions [133].

### Models of SC assembly in nematodes

The dual material properties of the CR invoke two models of SC assembly. The first assumes the CR assembles as a polymer, similar to cytoskeletal filaments, and that the main function of the axes is to localize and direct CR polymerization so that it occurs between the homologs. Elongation of the SC occurs through the piece-by-piece addition of subunits at the growing end. The CR’s ordered appearance [131] and its ability to exert force on chromosomes [122] are consistent with this model. Likening the CR to a cytoskeletal filament can account for the apparent cooperative assembly and is also consistent with the slower, rate-limiting nucleation and faster, processive elongation [73].

The second, “oozing” model, suggests that the CR acts as a liquid and that the axes act as a capillary for the CR to flow onto. Surface tension—the product of CR-axis interactions and of self-interactions between CR subunits—allows the CR to zip up the homologs, similar to the way liquids bring together and adhere pieces of glass or strands of hair [134]. Nucleation in this model reflects the initial formation of a separate phase by CR subunits. Phase separation may be accelerated in the vicinity of paired axes much like the condensation water vapors as dew on leaves. Elongation would reflect CR flow between the two chromosomes while bringing them together [14], with new subunits being recruited throughout the length of the SC [12,129,130].

Distinguishing between these models is not trivial since they are both consistent with our current knowledge of SC assembly and dynamics, such as assembly through distinct nucleation and elongation stages. Barring novel informative mutations or more precise measurements, physical models assuming either a liquid or a crystalline CR could be parameterized to account for SC assembly. The multiple roles of the SC and, consequently, the pleiotropic phenotypes of mutations in SC components, further limit the testing of the models. Finally, the two models may explain different stages of SC assembly, e.g., nucleation by condensation of CR material and elongation by filament-like polymerization (as was shown, e.g., for microtubule nucleation [135,136]).

Nonetheless, the need to distinguish between these models is an important challenge in research on the SC and on condensates more broadly, and it indeed constitutes a common criticism of the field [137]. Numerous cellular structures have been shown to exhibit liquid properties in vivo and their components exhibit similar properties in vitro [133]. However, in only a few cases has it been shown that a specific material state—e.g., a liquid—is crucial for the functions of the condensate.

### Future perspectives

Much work is needed to define CR-axis interaction interfaces. Ideally, this work will culminate in structural and biochemical characterization of the interfaces. As apparent from this survey, the rapid divergence of meiotic proteins entails that the molecular details of the interaction are unlikely to be easily extrapolated from one model organism to another. Nonetheless, progress made in one system is likely to inform the principles that underlie CR-axis interaction, as well as the implications of these interfaces for SC dynamics.

The recent insight into the dual material nature of the CR may provide an important conceptual and experimental framework. Recent work on other condensates involved developing physical models for their formation. These physical models stress the importance of multivalent interactions and predict some of the non-trivial emerging properties of phase-separated compartments, such as their ability to exert force on other cellular structures [134] or to form complex spatial patterns [138–141]. Along with the growing appreciation of the importance of condensation to cellular organization, there has been progress in developing techniques to reconstitute and analyze condensates [133]. The precise definition of the specific CR-axis interaction interfaces will enable their quantitative characterization (e.g., binding affinities). Such direct measurements of biophysical parameters, and analysis of the phenotypes resulting from disrupting such interfaces, will allow us to refine and test physical models of SC assembly.
